# Diosmin and Trolox Have Anti-Arthritic, Anti-Inflammatory and Antioxidant Potencies in Complete Freund’s Adjuvant-Induced Arthritic Male Wistar Rats: Roles of NF-κB, iNOS, Nrf2 and MMPs

**DOI:** 10.3390/antiox11091721

**Published:** 2022-08-30

**Authors:** Huda H. Shaaban, Walaa G. Hozayen, Amal K. Khaliefa, Ayman E. El-Kenawy, Tarek M. Ali, Osama M. Ahmed

**Affiliations:** 1 Department of Biochemistry, Faculty of Science, Beni-Suef University, Beni-Suef P.O. Box 62521, Egypt; 2 Department of Pathology, College of Medicine, Taif University, P.O. Box 11099, Taif 21944, Saudi Arabia; 3 Department of Physiology, College of Medicine, Taif University, P.O. Box 11099, Taif 21944, Saudi Arabia; 4 Physiology Division, Department of Zoology, Faculty of Science, Beni-Suef University, Beni-Suef P.O. Box 62521, Egypt

**Keywords:** diosmin, trolox, inflammation, rheumatoid arthritis, rats, oxidative stress

## Abstract

Rheumatoid arthritis (RA) is a chronic, progressive, autoimmune disease caused by a malfunction of the immune system. The aim of this study was to examine the anti-arthritic effects and suggest the mechanisms of actions of diosmin and trolox in male Wistar rats. Complete Freund’s adjuvant (CFA) was used to establish RA in the animals by subcutaneous injection of 100 µL CFA/rat into plantar region of right hind leg in two consecutive days. Diosmin and/or trolox were administered orally at a dosage of 20 mg/kg/day to CFA-induced arthritic rats for 2 weeks. The normal and arthritic control groups were orally given the same equivalent volume of a vehicle (1% carboxymethyl cellulose) in which treatment agents were dissolved. At the end of the experiment, blood samples were collected from the jugular vein for the detection of the total leukocyte count (TLC) and differential leukocyte count (DLC) in blood and the detection of rheumatoid factor (RF), anti-citrullinated protein antibodies (ACPA), tumor necrosis factor-α (TNF-α), interleukin-13 (IL-13), and interleukin-17 (IL-17) levels by enzyme-linked immunosorbent assay (ELISA), as well as markers of oxidative stress and the antioxidant defense system in serum. The right hind ankle regions of three rats from each group were dissected out and fixed in 10% neutral-buffered formalin for histological examination and the other three were kept at −30 °C for Western blot analysis of nuclear factor-kappa B (NF-κB) protein 50 (NF-κB p50), NF-κB p65, inducible nitric oxide synthase (iNOS), nuclear factor erythroid-2-related factor 2 (Nrf2), and matrix metalloproteinase (MMP)-1 (MMP-1), MMP-3, and MMP-9. The CFA injection was deleterious to the ankle joint’s histological architecture, manifesting as infiltration of inflammatory cells into the articular cartilage, hyperplasia of the synovium, and erosion of the cartilage. All these effects were ameliorated by diosmin and/or trolox, with the combined dose being the most effective. The two compounds significantly lowered the elevated serum levels of RF, ACPA, TNF-α, and IL-17, as well as other pro-inflammatory mediators, such as NF-κB p50, NF-κB p65, iNOS, MMP-1, MMP-3 and MMP-9. They also increased the levels of the anti-inflammatory cytokine, IL-13, and the cytoprotective transcription factor Nrf2. The compounds stimulated higher activities of antioxidants, such as glutathione, glutathione-S-transferase, catalase, and superoxide dismutase, and reduced lipid peroxidation in the serum of arthritic rats. In conclusion, diosmin, trolox, and their combination, which was the most potent, exerted anti-arthritic, anti-inflammatory and antioxidant effects by suppressing NF-κB signaling, inhibiting matrix metalloproteinases, and activating Nrf2.

## 1. Introduction

Rheumatoid arthritis (RA) is a debilitating, inflammatory, and autoimmune disease [1], which causes discomfort by damaging the bone, cartilage, and ligaments of the patient [2]. The disease is characterized by symmetric polyarthritis, synovitis, hyperplastic synovial cells, and erosion of the cartilage and bone [2,3]. About 0.5–1% of the global population is affected by RA, and its incidence in women is thrice that in men [4]. Also, its incidence significantly increases in people above 40 years old [5]. Despite the attributes of RA still being obscure [6], genetic, immunological, and environmental variables that contribute to its increased risk have been identified [7].

Complete Freund’s adjuvant (CFA)-induced arthritis exhibits pathophysiological features similar to human RA [8], making it a useful study model [9]. The progression of RA is evidently correlated with the serum levels of various cytokines, chemokines, and growth factors [10]. Cytokines are important in RA pathogenesis because they regulate a spectrum of inflammatory processes; the imbalance between pro- and anti-inflammatory cytokines in rheumatoid joints favors the induction of autoimmunity and chronic inflammation, leading to joint destruction [11]. Besides elevated levels of pro-inflammatory cytokines, high oxidative stress has been identified as a major cause of joint damage in RA [12]. Increased production of cytokines stimulates inflammatory cells, such as neutrophils and macrophages, to secrete reactive oxygen species (ROS) into the synovial fluid, mediating tissue injury [13].

Currently, conventional drug therapies prescribed for RA have side effects, prompting a global search for new therapeutics from natural sources [14,15,16,17,18]. These side effects depend on the type, dose and time of administration of the drugs, which are steroidal anti-inflammatory drugs (SAIDs), non-steroidal anti-inflammatory drugs (NSAIDs), and disease-modifying anti-rheumatic drugs (DMARDs). The corticosteroids side effects include ecchymosis, cushingoid features, parchment-like skin, leg edema, sleep disturbance, weight gain, epistaxis, glaucoma, depression, and hypertension [19]. The non-steroidal anti-inflammatory drugs could increase the risks of gastrointestinal and cardiovascular complications [20,21,22]. Methotrexate is the widely used and the most common DMARD and the first line therapies for RA have many side effects including nausea, flu-like symptoms, hepatotoxicity and hair loss [23] as well as mucositis, fever, infection, and purpura [24]. 

Diosmin (diosmetin-7-O-rutinoside) is an unsaturated glycoside distributed in various citrus fruits [25], obtained by dehydrogenating the equivalent flavanone hesperidin [26]. The antioxidant, antidiabetic, anti-inflammatory, and anticarcinogenic properties of this polyphenol have been demonstrated [25,27]. The capacity of diosmin to reduce the overexpression of nuclear factor–kappa B (NF-κB), tumor necrosis factor-α (TNF-α), cyclooxygenase-2 (COX-2), and inducible nitric oxide synthase (iNOS) has been correlated with its anti-inflammatory activities [26,28]. In addition, diosmin demonstrates the ability to cope with hepatic, renal, pulmonary, gastrointestinal, neurological, and myocardial injuries [29]. Diosmin is not immediately absorbed into the blood stream following oral administration; instead, it must first be digested by enterobacterial enzymes into its aglycone, diosmetin, which is then absorbed via the intestinal mucosa to the blood circulation [30]. Only diosmetin, the parent compound’s aglycone, is found in the plasma. The plasma elimination half-life of diosmetin is lengthy, ranging from 26 to 43 h. Diosmetin completely lacks urinary elimination, although its minor metabolites are, primarily in the form of glucuronic acid conjugates, removed in the urine. A metabolic pattern comparable to other flavonoids is confirmed by the presence of breakdown products including alkyl-phenolic acids [31]. Trolox (6-hydroxy-2,5,7,8-tetramethylchroman-2-carboxylic acid) is a water-soluble vitamin E (α-tocopherol) analog [32,33] with potent antioxidant and anti-inflammatory properties [34]. Trolox has little or no cytotoxic effects and can reduce cellular oxidative stress by half [35]. It has been shown to prevent lipid peroxidation (LPO), induced oxidative stress, and apoptosis by reducing the ROS output [36,37]. Trolox has advantages over vitamin E and is considered a more powerful scavenger of free radicals than its parent compound [38]. 

The aim of this study was to scrutinize the effects of diosmin and trolox on CFA-induced arthritis in male Wistar rats, with a focus on their mechanisms of action and the roles of NF-κB, iNOS, Nrf2 and MMPs.

## 2. Materials and Methods

### 2.1. Experimental Animals

Between January 2019 and January 2020, 40 male Wistar rats weighing 100–120 g and aged 6–7 weeks were studied at Beni-Suef University’s Faculty of Science in Egypt. The rats were obtained from the Egyptian Organization for Biological Products and Vaccines (VACSERA) animal house in Helwan, Cairo, Egypt. Animals were monitored for around 10 days before starting to rule out any concomitant infections. The animals were kept in polypropylene cages with well-ventilated stainless steel covers and were provided access to standard balanced food and water at their discretion at room temperature (25 °C ± 5 °C). The animals were weighed weekly during the experimental period. All animal procedures followed the IACUC rules at Beni-Suef University in Egypt (Ethical Approval Number: BSU/FS/2019/1). Every attempt was made to limit the number of animals as well as their misery and suffering.

### 2.2. Chemicals

Sigma Chemical Company provided CFA, a mineral oil (1 mg/mL) suspension of heat-killed *Mycobacterium tuberculosis*, diosmin, and trolox (Sigma Chemical Co., St Louis, MO, USA). 

### 2.3. Induction of Adjuvant-Induced Arthritis

A subcutaneous injection of 100 µL CFA/rat/day was administered into the plantar region of the rat’s right hind paw for two successive days to induce arthritis [14].

### 2.4. Animal Grouping

**Figure 1:**
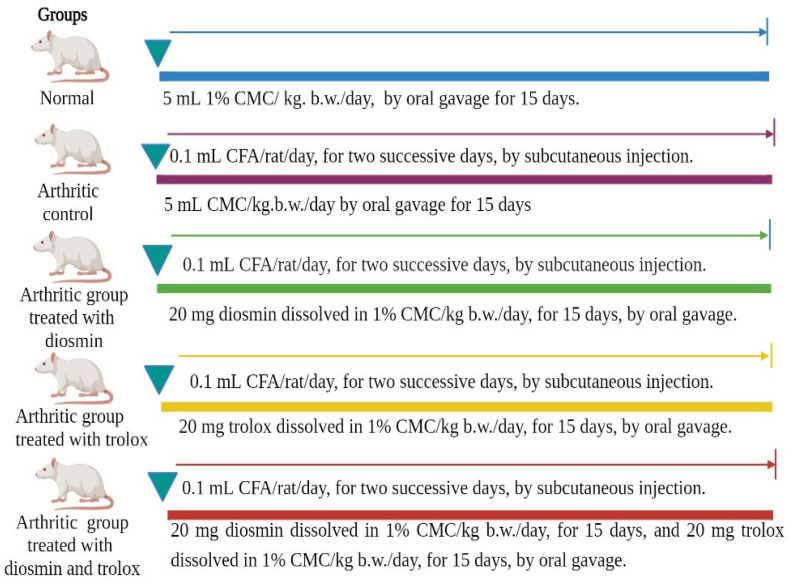
Experimental design and animal grouping.

After inducing RA by injecting CFA, Wistar rats were segregated into five groups (eight animals in each) designed as follows (Figure 1):

Group 1 (Normal control group): This group comprised normal rats orally administered the equivalent volume of the vehicle (1% carboxymethyl cellulose [CMC], 5 mL/kg/day) for 15 days.

Group 2 (Arthritic control group): This group consisted of arthritic rats who, similar to the normal control group, were orally administered the equivalent volume of the vehicle (1% CMC, 5 mL/kg/day) for 15 days. 

Group 3 (Arthritic group treated with diosmin): This group comprised arthritic rats administered a safe oral therapeutic dose of diosmin (20 mg/kg/day) [39] dissolved in 5 mL of 1% CMC for 15 days.

Group 4 (Arthritic group treated with trolox): This group comprised arthritic rats treated with a safe oral therapeutic dose of trolox (20 mg/kg/day) [40] dissolved in 5 mL of 1% CMC for 15 days.

Group 5 (Arthritic group treated with diosmin and trolox): This group comprised arthritic rats administered a safe oral therapeutic dose of diosmin and trolox (each dissolved in 1% CMC) for 15 days. 

At the end of the experiment, rats were euthanized by a cervical dislocation under diethyl ether inhalation anesthesia, and blood samples were obtained from the jugular vein. Part of the blood was collected in an anti-coagulated tube for the total leukocyte count (TLC) and differential leukocyte count (DLC). The other part of the blood was collected in a coagulating tube, centrifuged at 3000 rpm for 15 min, the clear non-hemolyzed supernatant sera were rapidly extracted, split into three aliquots for each individual animal, and stored at −20°C until required for further analyses. Three rats from each group had their right hind ankle region excised and fixed in 10% NBF (neutral-buffered formalin) for histological examination. The right hind ankles of three other rats from each group were stored at −30°C until they were used for Western blotting investigations.

### 2.5. Detection of Paw Edema

The swelling rate and paw edema in the different groups were measured by wrapping a thread around the circumference of the right paw just above the tarsal surface, as an indicator. A ruler was used to determine the length of the thread. Measurements were taken once a week after the adjuvant injection (on the 7th and 14th days) [16,18].

### 2.6. Histopathological Investigation

Following sacrifice and dissection, the right ankles were put into 10 percent neutral-buffered formalin (NBF) for 48 h for fixation before being transferred to the National Cancer Institute’s pathology department in Cairo, Egypt, for paraffin section preparation and hematoxylin and eosin staining (H&E).

Decalcification of the ankle was performed using a 10% formic acid solution. The ultimate point of decalcification was physically assessed with a surgical blade over the course of two weeks, with the formic acid solution being refilled twice weekly. Complete decalcification was followed by a graded ethanol series of dehydration before the specimen was embedded in paraffin wax. H&E was used to stain sections that were 5 µm thick.

### 2.7. Determination of Total Leukocyte Count (TLC) and Differential Leukocyte Count (DLC)

TLC and DLC were determined from a portion of the blood from the jugular vein collected in tubes containing ethylenediaminetetraacetic acid (EDTA) solution (50 μL of 15% EDTA for 2.5 mL of blood). For TLC, the cells were loaded onto a Neubauer hemocytometer slide using diluted gentian violet solution while the Giemsa stain was used to estimate DLC [17].

### 2.8. Enzyme-Linked Immunosorbent Assay (ELISA)

Serum levels of rheumatoid factor (RF), interleukin-17 (IL-17), interleukin-13 (IL-13), TNF-α, and anti-citrullinated protein antibodies (ACPA) were measured with specific ELISA kits, following the manufacturer’s instructions. RF and IL-17 were detected using ELISA kits purchased from CUSABIO (Houston, TX, USA). IL-13 was measured using an ELISA kit acquired from PicoKine^®^ (Boster Biological Technology, Pleasanton, CA, USA). TNF-α and ACPA were measured using ELISA kits provided by MyBioSource, Southern California, San Diego, CA, USA.

### 2.9. Evaluation of Oxidative Stress and Antioxidant Status

LPO, detected as malondialdehyde (MDA) levels and reduced glutathione (GSH) levels were estimated using the methods documented by Yagi [41] and Beutler et al. [42], respectively. The activities of antioxidant enzymes, such as glutathione-S-transferase (GST), superoxide dismutase (SOD), and catalase (CAT), were estimated using the methods documented by Mannervik and Guthenberg [43], Marklund and Marklund [44], and Cohen et al. [45], respectively.

### 2.10. Western Blot Analysis

The expressions of NF-κB protein 50 (NF-κB p50), NF-κB p65, iNOS, nuclear factor erythroid-2-related factor 2 (Nrf2), matrix metalloproteinase 1 (MMP-1), MMP-3, and MMP-9 were assayed in rat ankles by Western blotting, according to the method described by Harlow and Lane [46]. An ice-cold RIPA (radioimmunoprecipitation assay) buffer containing 0.1% PMSF (phenylmethylsulfonyl fluoride) was used to extract proteins from the three right hind ankle joints (Beyotime Biotechnology, Suzhou, China). SDS–PAGE (Sodium dodecyl sulfate–polyacrylamide gel electrophoresis) in 10% gels was utilized to separate equal amounts of proteins, which were subsequently transferred to PVDF (polyvinylidene difluoride) membranes. The PVDF membranes were probed overnight at 4 °C with 1^ry^ antibodies targeting the proteins of interest and β-actin (Santa Cruz Biotechnology, Inc., Dallas, TX, USA). The membranes were washed thrice using Tris-buffered saline with Tween 20 at room temperature. The blots were incubated with horseradish peroxidase-conjugated 2^ry^ antibodies (Santa Cruz Biotechnology, Santa Cruz, CA, USA) at a dilution of 1:5000 for 30 min. Finally, the blots were washed again and the chemiluminescent signal was detected on an X-ray film.

### 2.11. Statistical Analysis

The data are presented as mean ± standard error. The Statistical Package for Social Sciences (Chicago, IL, USA) was used to examine the data and the Duncan’s test was used to compare the results of different groups. For right hind paw circumference, a two-way analysis of variance (ANOVA) as well as a one-way ANOVA were performed. Statistical significance was defined as a *p* value less than 0.05.

## 3. Results

### 3.1. Gross Lesions of the Paw and Ankle Joint

**Figure 2:**
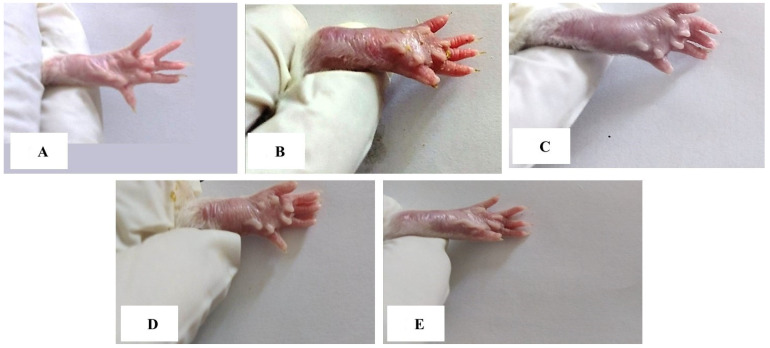
Gross morphology of right hind leg paw and ankle of rats showing swelling and inflammation in rats of different groups. (**A**): Ankle joint and paw of normal rat, (**B**): Ankle joint and paw of arthritic rat, (**C**): Ankle joint and paw of arthritic rats treated with diosmin, (**D**): Ankle joint and paw of arthritic rats treated with trolox, and (**E**): Ankle joint and paw of arthritic rats treated with diosmin and trolox.

Swelling, edema, and redness, which are external indicators for assessing the severity of arthritis, were observed in the right hind paws and ankles of CFA-induced arthritic rats. These gross lesions were reduced in the arthritic groups treated with diosmin and/or trolox, with the combined treatment being the most effective (Figure 2).

### 3.2. The Right Hind Paw Circumference

**Figure 3:**
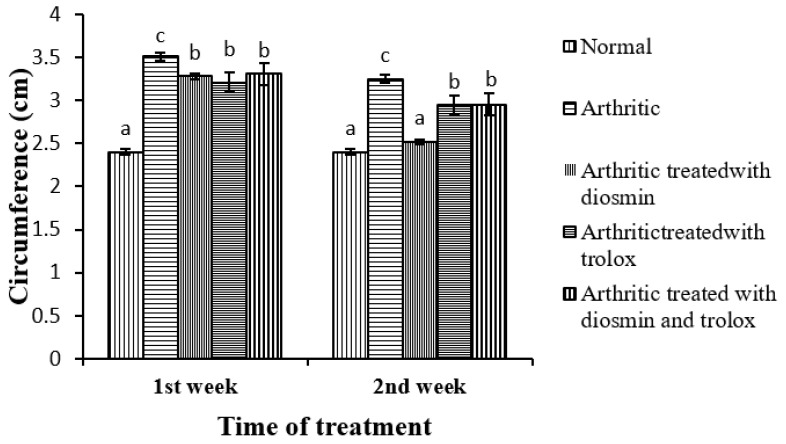
Effect of diosmin, trolox, and their combination on the circumference of the right hind paw in CFA-induced arthritic rats. Means with the same superscript symbol(s) are not statistically significant. a, b, and c refer to similarity or non-similarity between groups. Data are expressed as mean ± standard error and each group has a total of six individuals.

The effects of diosmin, trolox, and their combination on the circumference of the right hind paw in CFA-induced arthritic rats is depicted in Figure 3. The paw circumference significantly increased in the first and second weeks compared with the corresponding normal control group. Upon treatment with diosmin, trolox, or their combination, the paw edema significantly reduced by 7, 9, and 6% respectively in the first week and 22, 9, and 9%, respectively, in the second week, at the end of the trial, when compared with the arthritic control rats. The second week of treatment appeared to have a stronger beneficial effect on the hind paw circumference.

The one-way ANOVA indicated that the general effect was significant between groups (*p* < 0.05) throughout the experiment. The two-way ANOVA, on the other hand, depicted that the effect of treatment, time, and treatment–time interaction was significant at *p* < 0.05 (Table 1).

### 3.3. ELISA Results

Table 2 presents data showing the effects of diosmin, trolox, and their combination on the levels of serum RF, TNF-α, IL-13, IL-17, and ACPA in CFA-induced arthritic rats. In comparison to the control group, serum levels of RF, TNF-α, IL-17, and ACPA drastically increased in arthritic rats by 427, 256, 451, and 493%, respectively, while the IL-13 level significantly decreased by 129% (*p* < 0.05). When arthritic rats were given diosmin, trolox, or both, their elevated serum levels of RF, TNF-α, ACPA, and IL-17 were significantly downregulated, while the depleted level of IL-13 was significantly upregulated (*p* < 0.05). The combinatory effect was the most potent on RF, ACPA, TNF-α, IL-13 and IL-17 serum levels, recording percentages of −77, −67, −65, 116 and −72%, respectively. 

### 3.4. Oxidative Stress and Antioxidant Defense Markers

Table 3 displays the effects of diosmin, trolox, and their combination on serum LPO, GSH, GST, SOD, and CAT activity in CFA-induced arthritic rats. Arthritic rats had a 200% higher serum LPO level (*p* < 0.05) than normal rats. Upon treatment with diosmin, trolox, or both, serum LPO levels decreased by 38, 33, and 38%, respectively, when compared with arthritic rats. 

In CFA-induced arthritic rats, the serum GSH, GST, SOD, and CAT activity reduced by 53, 38, 77, and 75%, respectively, in comparison to normal rats. Treatment with diosmin, trolox, or both significantly increased (*p* < 0.05) the serum activities of all these antioxidant enzymes compared with arthritic rats. 

### 3.5. TLC and DLC

Table 4 shows the effects of diosmin, trolox, and their combination on the TLC and DLC in CFA-induced arthritic rats. Compared with the control rats, the arthritic rats displayed significantly higher leukocytosis (98%, *p* < 0.05) with considerable increases in the counts of neutrophils (156%), lymphocytes (163%), monocytes (147%), and eosinophils (117%). After treatment with diosmin, trolox, or both, the TLC dropped by 22, 25, and 20%, respectively, relative to arthritic rats. 

### 3.6. Western Blot Analysis

**Figure 4:**
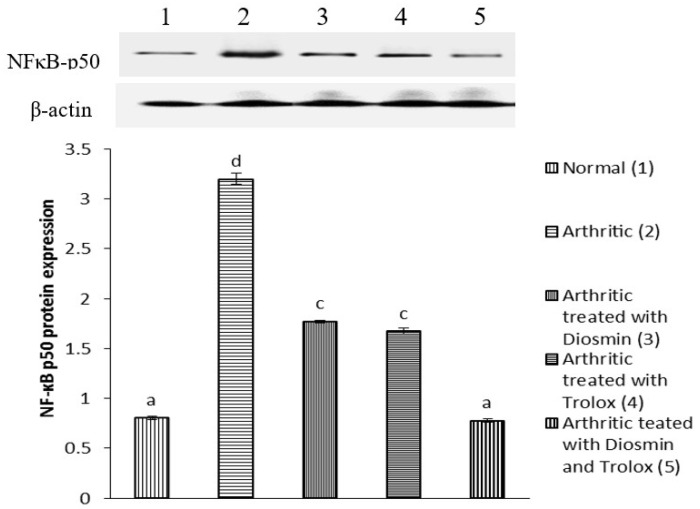
Effect of diosmin, trolox and their combination on NF-κB p50 protein expression level in CFA-induced arthritic rats. Means that have the same symbols (s) are not significantly different (*n* = 3).

**Figure 5:**
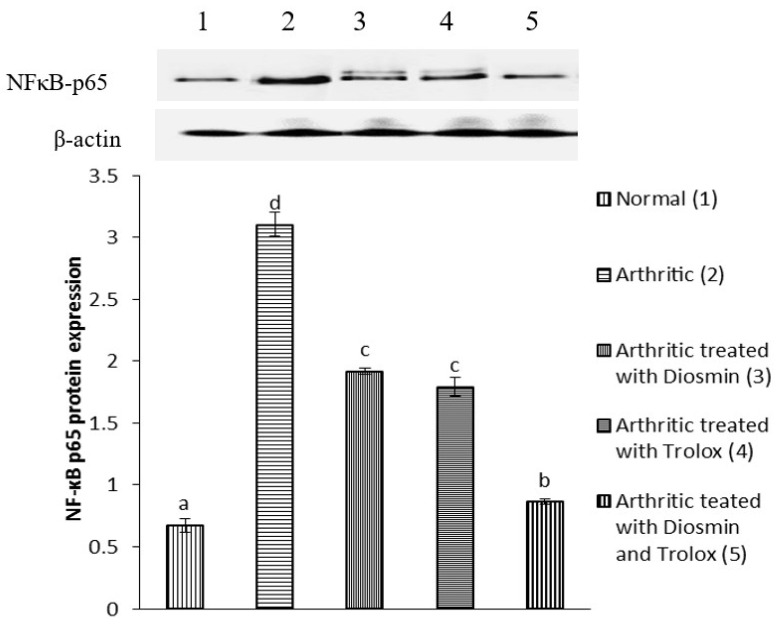
Effect of diosmin, trolox and their combination on NF-κB p65 protein expression level in CFA-induced arthritic rats. Means that have the same symbol (s) are not significantly different (*n* = 3).

**Figure 6:**
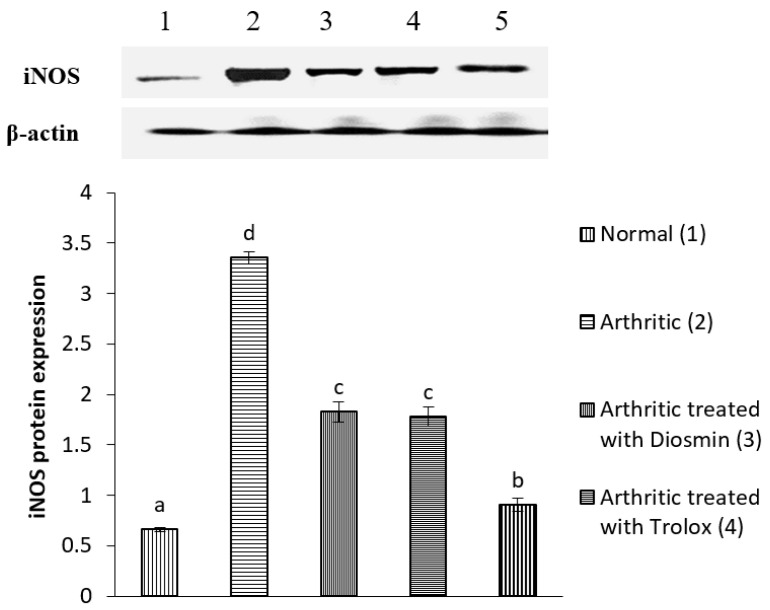
Effect of diosmin, trolox and their combination on iNOS protein expression level in CFA-induced arthritic rats. Means that have the same symbol (s) are not significantly different (*n* = 3).

**Figure 7:**
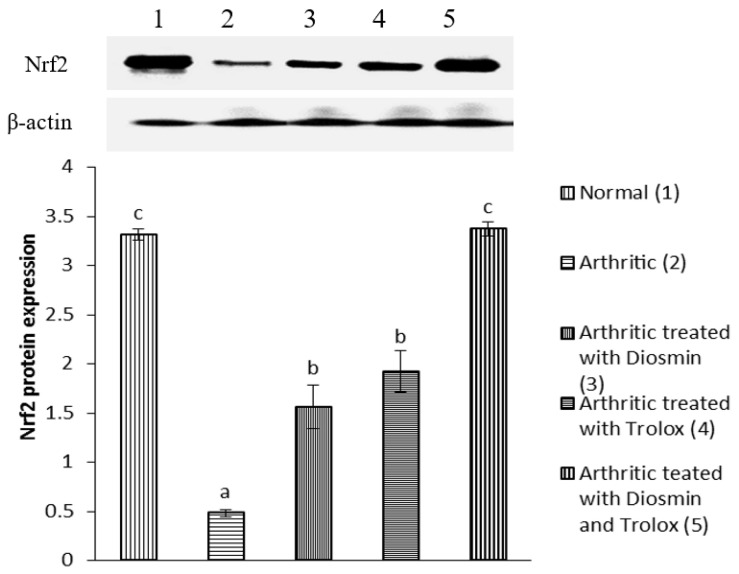
Effect of diosmin, trolox and their combination on Nrf2 protein expression level in CFA-induced arthritic rats. Means that have the same symbol (s) are not significantly different (*n* = 3).

**Figure 8:**
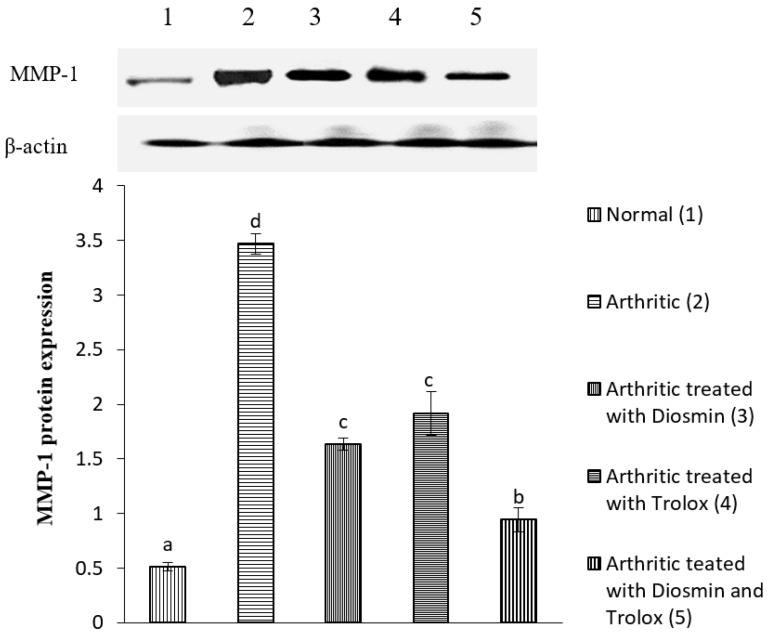
Effect of diosmin, trolox and their combination on MMP-1 protein expression level in CFA-induced arthritic rats. Means that have the same symbol (s) are not significantly different (*n* = 3).

**Figure 9:**
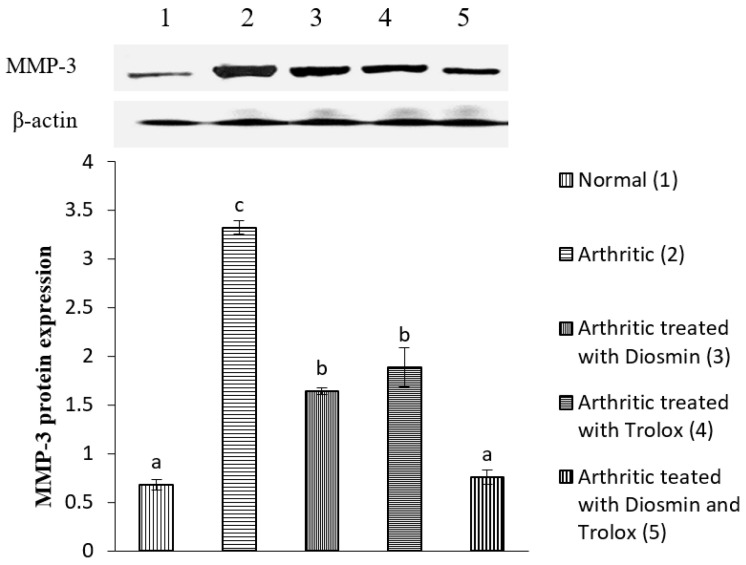
Effect of diosmin, trolox and their combination on MMP-3 protein expression level in CFA-induced arthritic rats. Means that have the same symbol (s) are not significantly different (*n* = 3).

**Figure 10:**
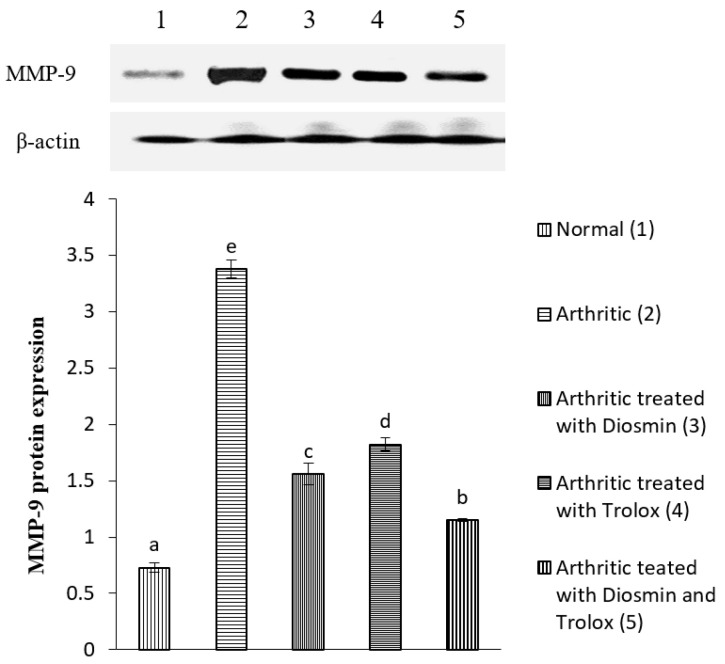
Effect of diosmin, trolox and their combination on MMP-9 protein expression level in CFA-induced arthritic rats. Means, that have the same symbol (s) are not significantly different (*n* = 3).

Figure 4, Figure 5, Figure 6, Figure 7, Figure 8, Figure 9 and Figure 10 illustrate the effects of diosmin, trolox, and both on the protein expression of NF-κB p50, NF-κB p65, iNOS, Nrf2, MMP-1, MMP-3, and MMP-9 in CFA-induced arthritic rats. 

The expression of NF-κB p50 and NF-κB p65 increased in arthritic rats by 298 and 361%, respectively, compared with control rats, while treatment with diosmin, trolox, and their combination reduced NF-κB p50 levels by 45, 48, and 76%, NF-κB p65 levels by 38, 42, and 72%, compared with arthritic rats.

When compared with the control group, the expression of iNOS increased by 409% in arthritic rats. Treatment with diosmin, trolox, or their combination decreased iNOS levels by 46, 47, and 73%, respectively, compared with arthritic rats.

Nrf2 expression was 86% lower in arthritic rats relative to control rats, while diosmin, trolox, or their combination therapy raised Nrf2 levels by 224, 298, and 601%, respectively, compared with arthritic rats.

The expression of MMP-1, MMP-3, and MMP-9 was higher in arthritic rats than in control rats by 576, 388, and 364%, respectively. Diosmin, trolox, or their combination, on the other hand, significantly reduced the levels of these matrix metalloproteinases (MMPs) when compared with arthritic rats (*p* < 0.05).

### 3.7. Histopathological Changes

**Figure 11:**
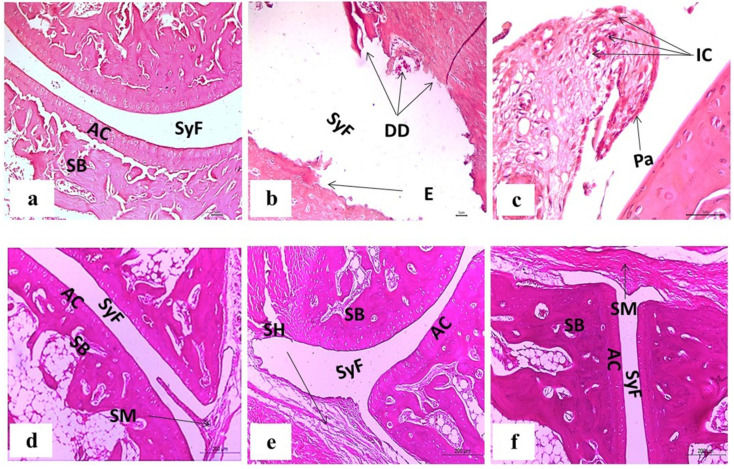
Photomicrographs of H&E-stained sections illustrating the effect of diosmin, trolox, and their combination treatments on the histological alterations in the ankle joints of arthritic rats. (**a**) Normal group. (**b**,**c**) CFA-induced arthritic group. (**d**) Arthritic rats given diosmin. (**e**) Arthritic rats given trolox. (**f**) Arthritic rats given diosmin and trolox. AC, SB, SyF, DD, E, Pa, IC, SM and SH stand for articular cartilage, sponge bone, synovial fluid, degenerative degradation, erosion, pannus formation, inflammatory cells, synovial membrane, and synovial hyperplasia, respectively.

The ankle joint sections from control rats, stained with H&E, revealed no signs of inflammation, with an intact articulating cartilage, synovial fluid, and sponge bone. The tissue sections from CFA-induced arthritic rats showed hyperplasia of the synovial membrane, with infiltration of a significant number of inflammatory cells, severe pannus development, erosion, and degenerative degradation of cartilage. Diosmin-treated arthritic rats had milder pathological arthritis, mild inflammation with an intact articular surface, mild synovial membrane projection, and mild congestion and edema of the periarticular tissue. Rats treated with trolox showed a nearly normal articular surface, focal mild hyperplasia in the synovial membrane without congestion, and no infiltration of inflammatory cells. Finally, rats administered a combination dose exhibited nearly normal articular surface and synovial membrane with no features of inflammation (Figure 11).

## 4. Discussion

RA is a persistent and progressive autoimmune illness caused when the immune system starts attacking healthy cells in the body, resulting in inflammation, swelling, and cartilage and bone destruction, especially in the joints [47]. The CFA-induced arthritis model is frequently used to evaluate the anti-arthritic activity of therapeutics because it closely resembles human arthritis, which is marked by joint swelling, infiltration of inflammatory cells, cartilage degradation, and bone loss [48].

In this study, the increased circumference of the right hind paw in arthritic rats was significantly decreased by treating them with diosmin and/or trolox. The change in paw edema has historically been used to assess an anti-inflammatory drug’s effectiveness in RA [34]. As indicated by the joint histology results in this and earlier studies, this drop in paw circumference signifies a decrease in swelling rate that can be related to a reduced edema, attenuated inflammatory processes, and reduced hyperplasia of the synovial tissue [14,15,16,17,18,49]. These findings could be attributed to the anti-inflammatory actions of diosmin and trolox [34,50]. 

RF, a circulating antibody against IgG, is a major serum marker for the diagnosis and prognosis of the severity of RA [51]. In the pathophysiology of RA, B lymphocytes are the source of RF and ACPA, which help create immune complexes and activate complement in the joints [52]. ACPAs are a highly distinguishing feature of RA patients. Anti-cyclic citrullinated peptide antibodies (ACCP) especially those against cyclic citrullinated peptide-2 (CCP2) have been proven to accurately predict both the onset of RA and the extent of concomitant joint damage [53]. When compared with RF, ACCP is more specific to RA and can be diagnosed before the disease progresses clinically [52].

Similar to the findings of Mohamed et al. [54], the serum RF and ACPA concentrations in CFA-induced arthritic rats were markedly elevated in our study, and treatment with diosmin and/or trolox significantly decreased them; the effect of the mixture was the most potent. The decrease in levels of RF and ACCP reflects a downregulation in the number of inflammatory cells, which agrees with Gokhale et al. [55], who emphasized the anti-inflammatory, antioxidant, and antiproliferative actions of flavonoids. Other studies have also reported the antioxidant, antimutagenic, antiapoptotic, and anti-inflammatory properties of diosmin [56] and the anti-inflammatory activity of trolox [34,57].

**Figure 12:**
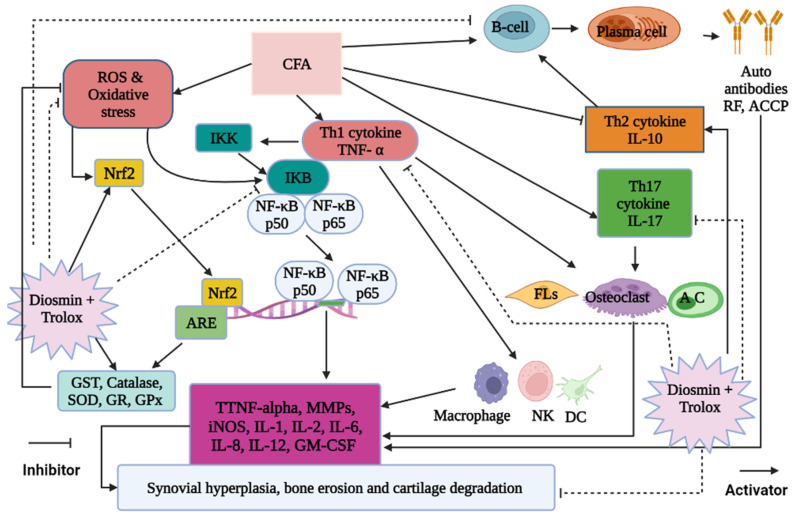
Schematic figure showing the mechanisms of actions of diosmin and trolox in arthritic rats.

The serum levels of TNF-α, IL-17, and IL-13 were measured to evaluate the anti-inflammatory effects of diosmin and/or trolox and their mechanisms of action. The pro-inflammatory cytokines TNF-α and IL-17 were found to be much higher in the serum of arthritic rats, whereas the anti-inflammatory cytokine IL-13 was found to be significantly lower. Due to these modifications, T helper 1 (Th1) and T helper 17 (Th17) cytokines prevail over Th2 cytokines [14,16,17,18,49,54]. The imbalance of cytokine production by Th1/Th2/Th17 cells is undoubtedly crucial in the pathophysiology of RA. The Th2 cytokine, IL-13, increases antibody-mediated immune responses [49]. In inflammatory arthritis models, IL-13 is a strong anti-arthritic cytokine that also suppresses cartilage degradation and osteoclastogenesis [58]. On the other hand, Th17 cells participate in the pathogenesis of autoimmune RA by releasing pro-inflammatory cytokines such as IL-17, IL-6, IL-21, IL-22, and TNF-α (Figure 12) [59]. In autoimmune disease models, the attenuation of Th17-secreted IL-17 cures tissue disease [60].

In the current study, arthritic rats showed reduced levels of IL-13, which is consistent with Ahmed et al. [61], who showed the same in the synovial fluid and tissues of RA patients. Treating arthritic rats with diosmin and/or trolox resulted in a considerable reduction in serum TNF-α and IL-17, and a significant elevation in serum IL-13, relative to the arthritic control rat. These results agree with previous reports demonstrating the anti-inflammatory and antioxidant properties of diosmin by inhibiting TNF-α and suppressing the activation of NF-κB [62,63]. The mixture of diosmin and trolox had the most potent effect, which may be due to the synergistic effects of treatment agents.

NF-κB, a heterodimer of the p65 and p50 subunits of the Rel protein family, is found in the cytoplasm of cells and maintained in an inactive state by binding to the IκBα inhibitory protein. IκBα is phosphorylated by IκB kinase in response to distress stimuli, such as ROS, inflammatory cytokines, and adjuvants like CFA (Figure 12) [64], which activates and releases NF-κB p65 and NF-κB p50 from their inhibitory unit. They migrate to the nucleus and regulate the production of pro-inflammatory cytokines such as TNF-α, IL-1, IL-6, and IL-17, as well as iNOS (Figure 12). Hence, the NF-κB p65 and p50 subunits are often used as markers for NF-κB activity [62,65].

In accordance with previous studies, our present investigation showed that CFA-induced arthritic rats had elevated levels of MMP-1, MMP-3, MMP-9, p65/p50 NF-κB complexes, and iNOS [66,67,68,69]. Contrarily, diosmin and/or trolox treatment significantly reduced the expression of all these proteins, with the combination dose being the most potent. These effects may be because the phenols suppress the nuclear transcription of NF-κB, which controls the production of iNOS [70]. In this way, diosmin might reduce ethanol-induced liver injury by suppressing the production of TNF-α, iNOS, and COX-1, and reducing the activation of NF-κB as reported by previous publication [71]. Thus, both diosmin and trolox probably exert their anti-inflammatory effects by inhibiting the activation of p65/p50 NF-κB complexes in arthritic joints, leading to the inhibition of inflammatory genes and a lower expression of TNF-α, iNOS, and MMPs (Figure 12). 

The white blood cells (leukocytes) play a crucial part in the body’s defense system. During inflammation, inflammatory cells, such as macrophages and neutrophils, as well as leukocytes and plasma components, travel to the site of infection or injury to eliminate threats [62]. In accordance with Ahmed et al. [14] and Ahmed et al. [17], the TLC and DLC data in our study demonstrated severe leukocytosis in the arthritic animals, along with elevated lymphocyte and monocyte counts. CFA, which is a mixture of attenuated *Mycobacterium tuberculosis* antigens and adjuvant, meant to promote an immune response in the host, could be the reason for this leukocytosis. Once the immune system has been stimulated, adjuvants cause inflammation due to leukocytic infiltration; as a result, the leukocyte count may rise, which correlates with arthritis severity. Treatment with diosmin and/or trolox significantly reduced the elevated TLC as well as the individual lymphocyte, monocyte, and neutrophil counts, proving that these therapies have an anti-inflammatory effect on arthritic rats [72,73].

Besides inflammation, oxidative stress is the other hallmark of RA. Multiple studies have indicated that people with RA have elevated oxidative stress along with increased inflammation [74]. In pathological or stressful situations, ROS overcomes the antioxidant systems, resulting in oxidative stress which irreversibly alters cell constituents, such as carbohydrates, proteins, and lipids, and disrupts normal cellular signaling [75]. Multiple chronic diseases, including arthritis, have been linked to an imbalance between prooxidant and antioxidant defense systems [76]. These cellular oxidants and altered antioxidants also activate the TNF-α, NF-κB, and Nrf2 pathways (Figure 12) [77].

In our present investigation, CFA-induced arthritic rats revealed a marked elevation in LPO accompanied with a reduction in the antioxidants GST, SOD, CAT, and GSH, which is consistent with Ahmed et al. [17] and Ahmed et al. [18]. Treatment with diosmin and/or trolox reduced oxidative stress mainly by scavenging free radicals, decreasing LPO levels, and increasing the levels of the aforementioned antioxidants. These results support the findings of Shalkami et al. [78] and Mahgoub et al. [79], which stated that the antioxidant effect of diosmin might be mediated both by scavenging ROS and by overexpressing antioxidant enzymes. Additionally, Wang et al. [80] have reported the antioxidant activity of trolox, which can scavenge radicals to directly neutralize the excessive ROS. trolox also inhibits LPO [32].

Nrf2 is a key modulator of cellular defense that counteracts the damage caused by oxidative stress [81]. It activates the basal and induced expressions of antioxidant response element (ARE)-dependent genes to control the physiological and pathophysiological outcomes of oxidant exposure (Figure 12) [82,83,84]. Thus, Nrf2 is a cyto-protective transcription factor involved in antioxidant and anti-inflammatory pathways in addition to its ability to prevent mitochondrial degradation by interacting with ARE (Figure 12) [85].

Along the lines of results reported by Xie et al. [86], the expression of Nrf2 in the ankle joint articular tissues of arthritic rats was significantly lower than that in normal rats. In arthritic animals, knocking out Nrf2 dramatically increased cartilage degradation and oxidative burden, while overexpressing it alleviated inflammation [87]. Treating arthritic rats with diosmin or trolox has been shown to increase Nrf2 expression levels [88], which agrees with Messier et al. [89], who discovered that trolox protected human and mouse primary alveolar type II cells from cigarette smoke damage through the Nrf2 pathway. The two compounds combined synergistically to most effectively upregulate the expression of Nrf2. 

Histopathological examinations revealed that arthritic rats showed an infiltration of inflammatory cells, hyperplastic synovial membranes, bone erosion, and degenerative degradation of cartilage. Diosmin treatment, on the other hand, considerably reduced joint damage and exhibited only a mild stage of these lesions, including an intact articular surface, sound meniscus, mild synovial membrane projection, and mild congestion and edema of the periarticular tissue. trolox-treated arthritic rats exhibited a nearly normal articular surface, focal mild hyperplasia in synovial membrane, and no congestion or infiltration of inflammatory cells. Finally, the combination treatment of diosmin and trolox resulted in a nearly normal articular surface and synovial membrane, without any features of inflammation. As a result, this study clearly demonstrated the effectiveness of the test compounds in suppressing inflammation inside tissues and inhibiting the paw swelling characteristic of arthritis.

## 5. Conclusions

The current investigation demonstrated that an anti-arthritic therapy comprising diosmin and/or trolox protected against oxidative stress and inflammation while ameliorating the histological effects on the articular joints of CFA-induced arthritic rats. The combined dose was the most effective. The anti-arthritic effects of these two compounds may be mediated by the suppression of NF-κB signaling, the activation of Nrf2 and antioxidant defense systems, and the repression of MMPs and pro-inflammatory cytokines. Clinical trials, however, are required to assess the efficacy and safety of this combination before it can be approved for use in humans.

## Figures and Tables

**Table 1:** Effect of treatment, time and their interactions on paw circumference in rats (one-way and two-way ANOVA).

	Source of Variance	Sum of Squares	D.F.	Mean Squares	F Ratio	*p* Value
One-way ANOVA	General effect In between groups	9.014	9	1.002	37.771	*p* < 0.05
	Within groups	1.326	50	0.027		
	Total	10.34	59			
Two-way ANOVA	Treatment	6.467	4	1.617	60.966	*p* < 0.05
	Time	1.647	1	1.647	62.1	*p* < 0.05
	Treatment-Time	0.901	4	0.225	4.494	*p* < 0.05

**Table 2:** Effect of diosmin and/ or trolox on serum RF, TNF-α, IL-13, IL-17 and ACPA level of CFA- induced arthritic animals.

Groups	RF (µIU/L)	% Change	ACPA (ng/mL)	% Change	TNF-α (pg/mL)	% Change	IL-13 (pg/mL)	% Change	IL-17 (pg/mL)	% Change
Normal	17.9 ± 1.36 ^a^	-	1.50 ± 0.12 ^a^	-	31.5 ± 1.77 ^a^	-	158 ± 4.79 ^c^	-	18.1 ± 1.35 ^a^	-
Arthritic	94.4 ± 1.60 ^c^	427	8.90 ± 0.34 ^d^	493	112 ± 4.45 ^d^	256	69.1 ± 1.06 ^a^	−129	99.7 ± 3.27 ^d^	451
Arthritic treated with Diosmin	37.7 ± 2.04 ^b^	−60	4.70 ± 0.16 ^c^	−47	56.3 ± 5.28 ^b^	−50	123 ± 1.42 ^b^	78	53.7 ± 2.73 ^c^	−46
Arthritic treated with Trolox	36.0 ± 6.49 ^b^	−62	4.00 ± 0.46 ^c^	−56	86.8 ± 3.12 ^c^	−23	122 ± 9.80 ^b^	77	48.2 ± 1.68 ^c^	−52
Arthritic treated with Diosmin and Trolox	21.9 ± 1.94 ^a^	−77	2.90 ± 0.36 ^b^	−67	39.0 ± 2.14 ^a^	−65	149 ± 2.14 ^c^	116	27.6 ± 1.05 ^b^	−72

Data are expressed as mean ± standard error. Each group has a total of six individuals. In the same column, means with the same superscript symbol(s) are not statistically significant. Comparisons of the arthritic group with the normal and arthritic treatment groups with the arthritic group yielded percentage changes (% change).

**Table 3:** Effect of diosmin and/ or trolox on antioxidant defense and oxidative stress markers in the serum of CFA-induced arthritic animals.

Groups	LPO (nmole MDA/100 mL/hr)	% Change	GSH (nmole/100 mL)	% Change	GST (U/mL)	% Change	SOD (U/mL)	% Change	CAT (U/mL) × 10^2^	% Change
Normal	0.7 ± 0.47 ^a^	-	13.2 ± 1.65 ^b^	-	147.3 ± 7.94 ^c^	-	4.4 ± 0.27 ^c^	-	2.00 ± 0.12 ^b^	-
Arthritic	2.1 ± 0.10 ^c^	200	6.2 ± 0.50 ^a^	−53	77.1 ± 3.76 ^a^	−48	1.0 ± 0.09 ^a^	−77	0.50 ± 0.07 ^a^	−75
Arthritic treated with Diosmin	1.3 ± 0.12 ^b^	−38	11.5 ± 0.83 ^b^	86	109.9 ± 4.67 ^b^	43	2.5 ± 0.15 ^b^	150	1.50 ± 0.09 ^b^	200
Arthritic treated with Trolox	1.4 ± 0.07 ^b^	−33	10.3 ± 0.75 ^b^	66	115.1 ± 13.31 ^b^	49	3.1 ± 0.31 ^b^	210	1.70 ± 0.35 ^b^	240
Arthritic treated with Diosmin and Trolox	1.3 ± 0.02 ^b^	−38	12.5 ± 1.05 ^b^	102	108.6 ± 8.91 ^b^	41	2.9 ± 0.31 ^b^	190	1.65 ± 0.06 ^b^	230

Data are expressed as mean ± standard error. Each group has a total of six individuals. In the same column, means with the same superscript symbol(s) are not statistically significant. Comparisons of the arthritic group with the normal and arthritic treatment groups with the arthritic group yielded percentage changes (% change).

**Table 4:** Effect of diosmin and/ or trolox on TLC and DLC of CFA-induced arthritic animals.

Groups	TLC (Cell × 10^3^/mm^3^)	% Change	Neutrophil	% Change	Lymphocyte	% Change	Monocyte	% Change	Eosinophil	% Change
Normal	5.78 ± 0.26 ^a^	-	1274.3 ± 32.50 ^a^	-	2991.0 ± 147.4 ^a^	-	117 ± 12.3 ^a^	-	53.7 ± 3.7 ^a^	-
Arthritic	11.4 ± 0.49 ^c^	98	3266.3 ± 247.9 ^c^	156	7865.8 ± 354.3 ^c^	163	289 ± 24.4 ^c^	147	116.8 ± 7.3 ^c^	117.5
Arthritic treated with Diosmin	8.88 ± 0.28 ^b^	−22	2272.3 ± 137.4 ^b^	−30.4	5793.7 ± 226.8 ^b^	−26.3	227.7 ± 9.6 ^b^	−21.2	88.7 ± 2.8 ^b^	−24.1
Arthritic treated with Trolox	8.60 ± 0.66 ^b^	−25	2161.2 ± 204.6 ^b^	−33.8	5588 ± 260.9 ^b^	−30	130 ± 15.1 ^a^	−55	88.3 ± 5.1 ^b^	−24.4
Arthritic treated with Diosmin and Trolox	9.12 ± 0.24 ^b^	−20	2958.2 ± 70.00 ^c^	−9.4	5440 ± 322.6 ^b^	−30.8	125.2 ± 26.9 ^a^	−56.7	94.8 ± 6.1 ^b^	−18.8

Data are expressed as mean ± standard error. Each group has a total of six individuals. In the same column, means with the same superscript symbol(s) are not statistically significant. Comparisons of the arthritic group with the normal and arthritic treatment groups with the arthritic group yielded percentage changes (% change).

## Data Availability

All data are included in the article.

## Abbreviations

ACPA: Anti-citrullinated protein antibody; ACCP: anti-cyclic citrullinated peptides; AIA: Adjuvant induced arthritis; B cells: B lymphocytes; CAT: Catalase; CFA: Complete Freund’s adjuvant; CMC: Carboxymethyl cellulose; COX-2: Cyclooxygenase-2; COX-1: Cyclooxygenase 1; FLS: Fibroblasts like synoviocytes; GSH: Reduced glutathione; H_2_O_2_: Hydrogen peroxide; HOCl-: Hypochlorous acid; IL: Interleukin; IL-1β: Interleukin-1β; iNOS: Inducible nitric oxide synthase; LPO: Lipid peroxidation; MMPs: Matrix metalloproteinases; MMP-1: Metalloproteinase 1; MMP-2: Metalloproteinase 2; MMP-3: Metalloproteinase 3; MMP-9: Metalloproteinase 9; NF: Nuclear factor; NF-κB: Nuclear transcription factor–kappa B; NF-κB p65: Nuclear factor–kappa B protein 65; NF-κB p50: Nuclear factor–kappa B protein 50; NO: Nitric oxide; NK: Natural killer cell; Nrf2: Nuclear factor erythroid derived-2-related factor 2; O2^–^: Super oxide anion; OH^−^: Hydroxyl radical; PGE2: prostaglandins E2; PMSF: Phenylmethylsulfonyl fluoride; PVDF: Polyvinylidene difluoride; RF: Rheumatoid factor; RA: Rheumatoid arthritis; RNS: Reactive nitrogen species; RIPA: radioimmunoprecipitation assay; ROS: Reactive oxygen species; SDS-PAGE: Sodium dodecyl sulfate-polyacrylamide gel electrophoresis; sIL-17R: Soluble interleukine-17 receptors; SOD: Superoxide dismutase; T cells: T lymphocytes; TNF-α: Tumor necrosis factor alpha; TXA2: Thromboxane A2.
